# Socio-demographic and household attributes may not necessarily influence malaria: evidence from a cross sectional study of households in an urban slum setting of Chennai, India

**DOI:** 10.1186/s12936-017-2150-z

**Published:** 2018-01-05

**Authors:** Shalu Thomas, Sangamithra Ravishankaran, Aswin Asokan, N. A. Johnson Amala Justin, T. Maria Jusler Kalsingh, Manu Thomas Mathai, Neena Valecha, Alex Eapen

**Affiliations:** 1 IDVC Field Unit, ICMR-National Institute of Malaria Research, NIE Campus, 2nd Main Road, TNHB, Ayapakkam, Chennai, 600 077 India; 2 Department of Zoology, Madras Christian College, Tambaram, Chennai, 600 059 India; 3 ICMR-National Institute of Malaria Research (ICMR), Sector 8, Dwarka, New Delhi, 110 077 India

**Keywords:** Urban malaria, Breeding habitats, Overhead tanks, Occupation, Approachability

## Abstract

**Background:**

Household and environmental factors are reported to influence the malaria endemicity of a place. Hence, a careful assessment of these factors would, potentially help in locating the possible areas under risk to plan and adopt the most suitable and appropriate malaria control strategies.

**Methods:**

A cross-sectional household survey was carried out in the study site, Besant Nagar, Chennai, through random sampling method from February 2014 to February 2015. A structured interviewer-administered questionnaire was used to assess selected variables of demography, structural particulars of a household, usage of repellents, animals on site, presence of breeding habitats and any mosquito/vector breeding in the household, malaria/vector control measures undertaken by government in each houses. The data was collected through one to one personal interview method, statistically analysed overall and compared between the households/people infected with malaria within a period of 1 year and their non-infected counterparts of the same area.

**Results:**

Presence of malaria was found to be significantly associated with the occupation, number of inhabitants, presence of a separate kitchen, availability of overhead tanks and cisterns, immatures of vector mosquitoes, presence of mosquito breeding and type of roof structures (p < 0.05). However, age, gender, usage of repellents, animals on site, number of breeding habitats or detection of vector breeding did not significantly associate with the malaria incidence/prevalence.

**Conclusions:**

The survey revealed various demographic, household and environmental factors likely to associate with the malaria incidence/prevalence in an urban slum of Chennai. The socio-demographic and household variables have revealed disparities in malaria infection from the present cross sectional study. The absence of significant association with many parameters indicates the probable role of other confounding factors which influence the malaria prevalence.

## Background

The malaria endemicity of any area corresponds to the simultaneous presence of vector, circulating parasite, susceptible hosts (humans) and the contact between these three components [1, 2]. Though the presence of these three factors are the major driving force of malaria, the successful and continuous connections between these three components are the keys for disease transmission. Geo-climatic factors such as temperature, moisture, water quality determine the presence of *Anopheles* breeding sites, vector densities, adult mosquito survival rate, longevity and vector capacity [3].

The potential impacts of environmental and demographic factors on malaria resurgence and local transmission are becoming more critical points of discussion in recent times. Multi-disciplinary analyses of malaria control and studies to understand the increase in malaria incidence/prevalence has reported the importance of social, economic and other contextual variables. The data regarding social, cultural, economic and environmental factors can form a robust platform in mapping of risk models. Utilizing these factors, generally inbuilt in the population structure, however, are often underestimated and analysing their potentialities in malaria endemicity of a region will be useful for long-term plans. Moreover, it expands the current understanding of the multi-factor interactions involved in the malaria incidence/prevalence and disease transmission [4].

The Municipal Corporation of Chennai implements Urban Malaria Scheme (UMS) which consists of anti-larval activities such as larvicidal application and fogging besides, anti-parasitic measures [5]. Factors associated with perennial transmission and malaria prevalence in an area specific field settings are extremely important in targeting malaria pockets (hot spots) and then subsequent control measures. Unfortunately, in this regard only a very few studies, have been carried out in India [6]. It has been reported that urban malaria transmission varied not only on vector abundance, but also to a few additional factors which includes socio-economic factors, breeding sites as well as local malaria interventions [7]. Urban malaria control have become a challenge due to the lack of inter-sectoral coordination, poor planning, mosquito control is usually practised rather than species sanitation, acute water storage and erratic water supply in highly dense areas, water storage in a variety of containers, inadequate man power to tackle vector control operation and parasite surveillance, empirical and incomplete treatment, noncompliance to primaquine treatment for 14 days in *Plasmodium vivax*, financial constraints besides, municipal/corporation byelaws neither amended nor been practiced.

It is known that socio-demographic as well as socio-economic factors influence the success of malaria intervention strategies in the community level [6]. Thus, assessing various socio-demographic, household and environmental factors associated with the malaria incidence/prevalence in an endemic region would potentially help in locating the possible areas under risk (hotspot) and to deploy the most sustainable and appropriate control strategies. Hence the present study was aimed to assess the various socio-demographic factors associated with the malaria incidence/prevalence among residents in an urban slum, Besant Nagar, Chennai, endemic for malaria with perennial transmission.

## Methods

### Study site and survey

**Fig. 1:**
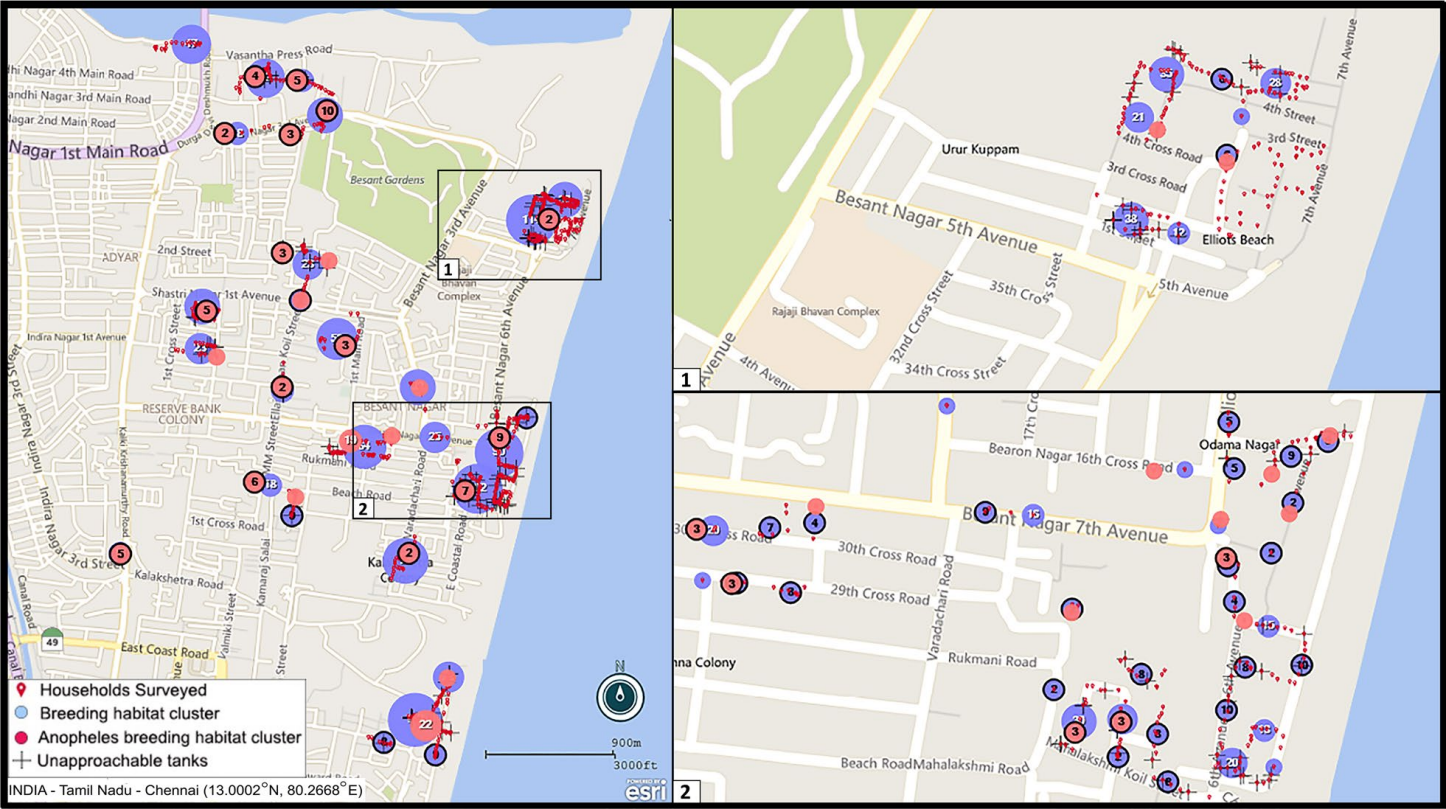
Study area with breeding habitats and the malaria incidence/prevalence

**Fig. 2:**
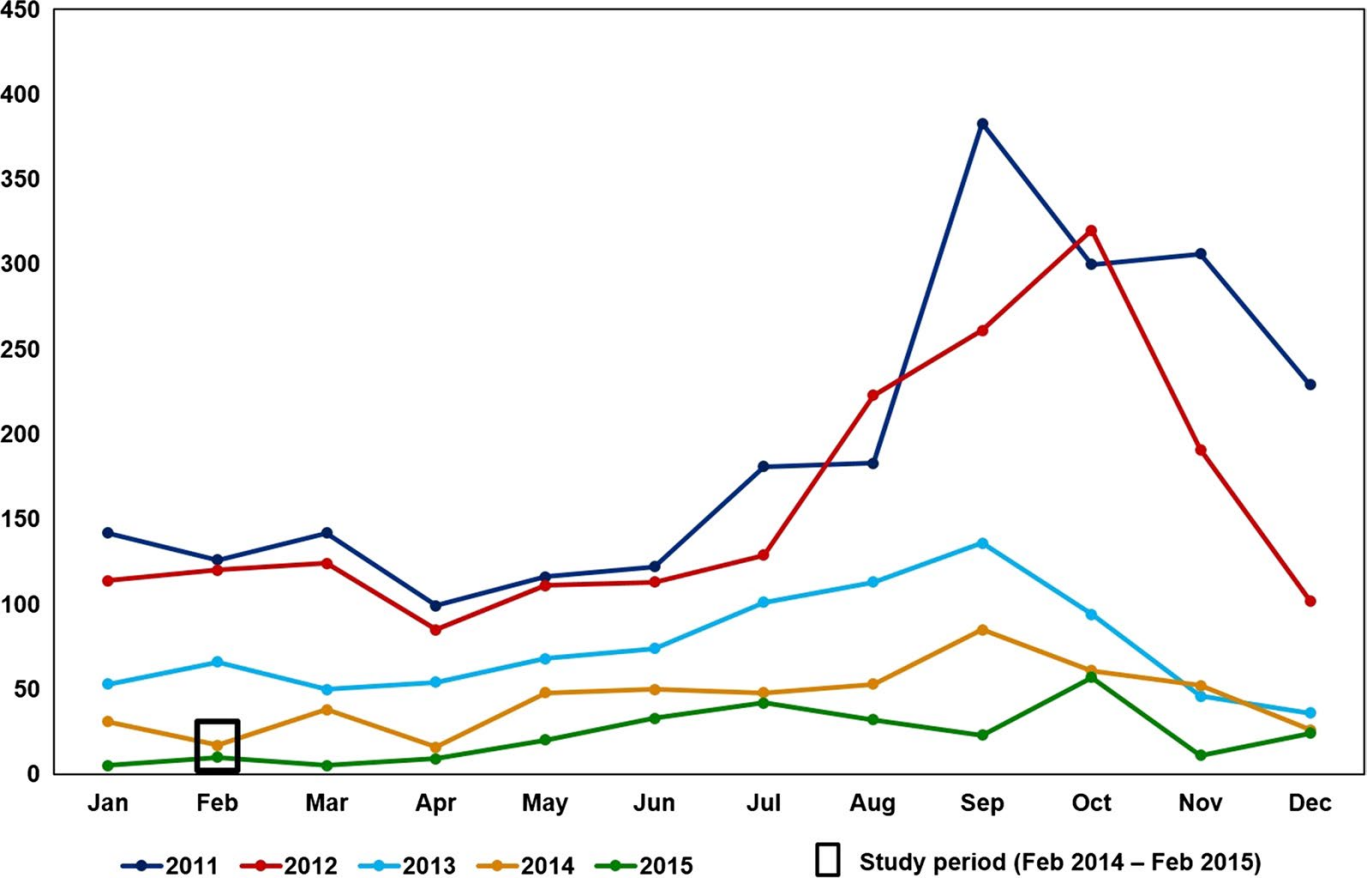
Malaria prevalence of the study site with demarcation of the study period

**Fig. 3:**
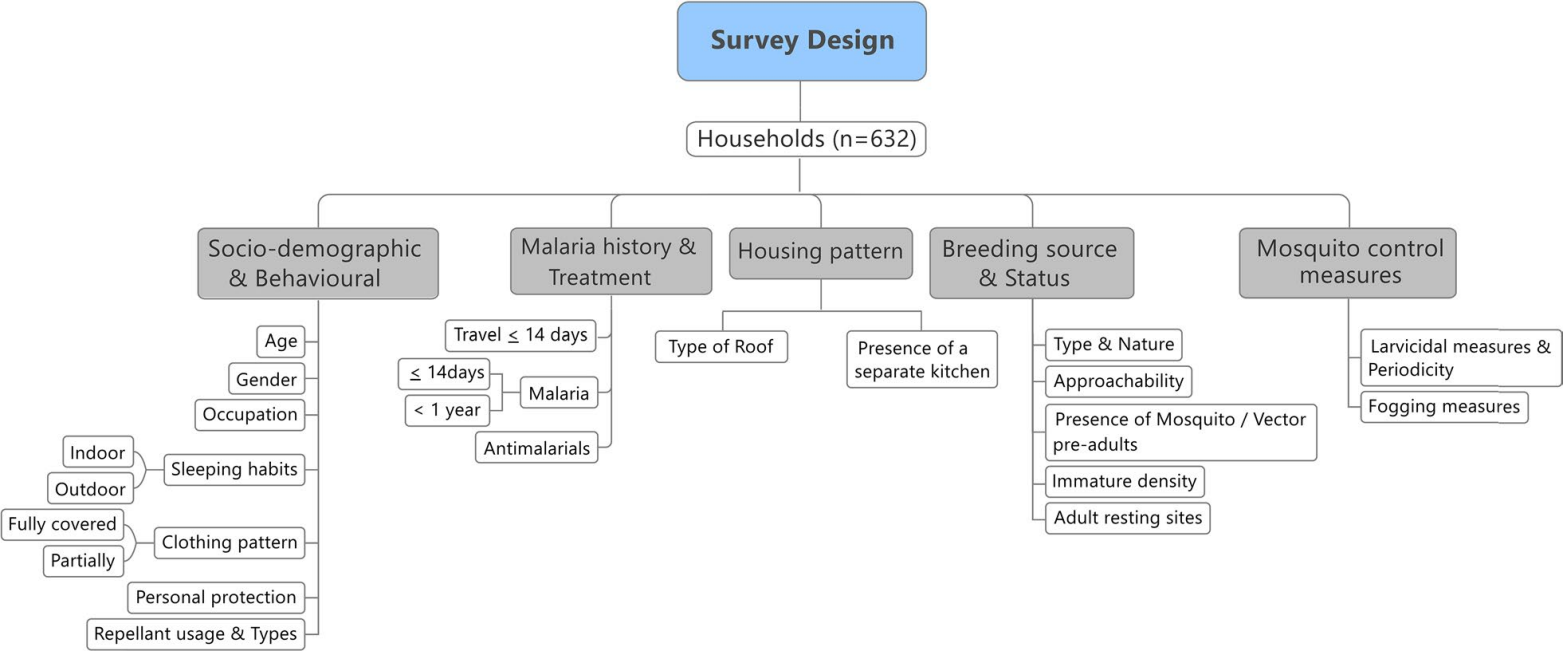
Design of the household survey on socio-demographic attributes of malaria

The study site, Besant Nagar (13.0002˚N, 80.2668˚E) is in the south-eastern part of Chennai (Fig. 1), characterized by its meso-endemic perennial transmission of malaria (Fig. 2), predominantly *Plasmodium vivax*, by the Asiatic urban malaria vector, *Anopheles stephensi* [5, 8]. A random, cross-sectional household survey was carried out across the study site from February 2014 to February 2015 in order to understand the association between various socio-demographic, household as well as environmental parameters and the local malaria incidence/prevalence. An overview of the parameters/attributes included in the survey is presented in Fig. 3. A structured interviewer-administered questionnaire was prepared to assess the malariogenic conditions. The questionnaire included queries on selected variables of demography, household information, usage of repellents, breeding habitats or sources, vector breeding in the household and vector control measures undertaken. The data was collected through one to one personal interview method, statistically analysed overall and compared between people who were affected with malaria and uninfected subjects of the same area within a period of 1 year.

### Study population and data collection

The study population included inhabitants of houses from the urban slum area of Besant Nagar. Households were randomly selected and approached for their willingness to participate. Any household member who is 18 years or above was interviewed and the details were recorded in a questionnaire based survey form. All the 632 households enrolled were inhabitants of the study site for the past 1-year. The variables associated with malaria and transmission were analysed depending on the malaria cases within the study period. Only those parameters which were assumed to have remained constant throughout the study period were selected. The breeding habitats were also checked for the presence/absence of mosquito immatures, in order to ascertain the vector breeding potential of each household.

### Socio-demographic characteristics

Socio-demographic information collected included age, gender and occupational status of household members. The subjects were queried on the malaria infection of any member of the household within a period of 1 year. They were asked about the actual period they contracted the infection, treatment seeking behaviour (whether private or government hospitals/clinics), and on the compliance of the anti-malarial treatment administered. Further, the households were categorized based on the type of roof structures like concrete, tiled, asbestos and thatched which depends on the preferred resting sites of vector mosquitoes. Also, the houses were checked for the presence of a separate kitchen, which prevents the heat dissipated due to cooling and also animals belonging to the site/particular house.

### Preventive measures against mosquito bites and vector control management practices

Queries were made on the preference of mosquito repellents used in each household. Subjects were enquired about the type of the repellents used, like coils, vaporizers, mats, various repellent creams, mosquito bats and mosquito nets used in the household. Since the usage of repellent creams, mosquito bats and mosquito nets widely varied among the household members, they were excluded from the analysis. Coils, vaporizers and mats were observed to be used by the entire household rather than individual members, and were included in the analysis. Subjects were also queried about the frequency of anti-vector measures carried out by the vector control programme personnel such as application of larvicides and fogging operation.

### Vector breeding habitats

The households were surveyed for the presence or absence of a potential vector breeding source/habitat like Overhead tanks, wells, stored water containers (plastic pots, plastic containers and mud pots), cisterns (barrels or drums), inside tanks, underground tanks or sumps. The number and type of breeding habitats were recorded. Further, if any of the habitats were observed with the presence of mosquito breeding, the details of the mosquito genera, instar wise count and presence of vector immature were recorded. The data of inaccessible breeding habitats, their types and number was documented separately. Figure 1 illustrates the study area with breeding habitats including unapproachable ones together with the malaria incidence/prevalence during the study period.

### Data management and analysis

The responses from the household members were entered by individual trained interviewers in the field. However, once completed, the questionnaire forms were re-checked for errors and ensured completion. Data were then entered into a Microsoft Excel 2010 spread sheet. Subsequently, age of the participants was grouped into four categories for analysis (1–4, 5–9, 10–17, ≥ 18) and households were categorized based on the number of inhabitants (≤ 3 and > 3). Data cleaning was made with the field collected questionnaire formats for any mistakes/errors or incorrect entries. After the completion of survey, summary scores were computed using descriptive statistics of IBM SPSS version 21. Among the 632 households surveyed, five households did not have any well-defined roof types and so, they were excluded from the roof type analysis. Categorical variables were compared using the Chi square analysis and a significance level of p  <  0.005 was set for all statistical tests.

## Results

### Outcome of the household survey

In total, 2471 people from 632 households were included in the survey. 90 people from 71 households were reported to have malaria during the past 1 year and thus 12 (16.9%) houses had more than one person infected with malaria during the past 1 year of survey. The prevalence of malaria in the study site was found to be 4 (95% CI 3.0–4.5). All the people who reported to have had previous episodes of malaria sought treatment, irrespective of the treatment facility like government or private sector. However, a potential recall bias was observed for the question whether they have completed the course of anti-malarial received. A total of 1137 breeding habitats were surveyed, out of which 119 were found to be positive for *Anopheles stephensi*. Out of 71 households with malaria patients, 57 houses had at least one of the mosquito breeding habitats (irrespective of mosquito species). The characteristics of total population, malaria positive subjects and households as well as their non malarious counterparts were calculated (Table 1). Breeding habitats surveyed were categorized based on their types, its approachability/accessibility for the routine immature surveillance and larvicidal treatment together with presence of mosquito breeding (Table 2).

**Table 1:** Socio-demographic and household attributes and the percent population with malaria for the attributes

Socio-demographic attributes	Categories	Number of surveyed population positive for the attributes (%)	Number of malaria patients positive for the attributes (%)	Number of non-malaria patients positive for the attributes (%)	χ^2^, P value
		n = 2471	n = 90	n = 2381	
Gender	Male	1256	48 (3.8)	1208 (96.2)	
	Female	1215	42 (3.5)	1173 (96.5)	
Age group	1–4 years	122	1 (0.8)	121 (99.2)	
	5–9 years	155	5 (3.2)	150 (96.8)	
	10–17 years	237	15 (6.3)	222 (93.7)	
	Above 18 years	1957	69 (3.5)	1888 (96.5)	
Occupation (away from home during day time-going either to work place or to educational institutions)	Yes	1540	65 (4.2)	1475 (95.8)	3.898, 0.048
	No	931	25 (2.7)	906 (97.3)	

Household attributes	Categories	Number of surveyed households positive for the attributes (%)	Number of malarious households positive for the attributes (%)	Number of non-malarious households positive for the attributes (%)	χ^2^, P value
		n = 632	n = 71	n = 561	
Vector control and prevention measures	Fogging	419	49 (11.7)	370 (88.3)	
	Antilarval	521	63 (12.1)	458 (87.9)	
Repellent use	Houses which use at least one type of repellent (coils, vaporizers, mats)	346	34 (9.8)	312 (90.2)	
	Houses using coil	49	9 (18.4)	40 (81.6)	
	Houses using mat	8	1 (12.5)	9 (112.5)	
	Houses using vaporizer	289	24 (8.3)	265 (91.7)	
Size of the family/household and type of resident	Houses with inhabitants ≤ 3	227	9 (4)	218 (96)	18.772, < 0.001
	Houses with inhabitants > 3	405	62 (15.3)	343 (84.7)	
	Residing in concrete houses	473	37 (7.8)	436 (92.2)	232.48, < 0.001
	Residing in thatched houses	64	14 (21.9)	50 (78.1)	
	Residing in tiled houses	12	2 (16.7)	10 (83.3)	
	Residing in asbestos houses	78	18 (23.1)	60 (76.9)	
	Presence of a separate kitchen	450	41 (9.1)	409 (90.9)	12.506, 0.002
	Presence of any animals/pets around household	202	28 (13.9)	174 (86.1)	
Presence and nature of breeding habitat	Presence of breeding source	552	57 (10.3)	495 (89.7)	
	Presence of breeding source with any mosquito breeding	143	9 (6.3)	134 (93.7)	4.524, 0.033
	Presence of breeding source with vector immatures	101	3 (3)	98 (97)	8.232, 0.04
	Presence of breeding source with at least one unapproachable habitat	455	45 (9.9)	410 (90.1)	
	Presence of OHT	463	38 (8.2)	425 (91.8)	15.907, < 0.001
	Presence of cisterns	34	9 (26.5)	25 (73.5)	8.365, 0.04

**Table 2:** Characteristics of breeding habitats, proportions of mosquito/vector breeding and access for vector surveillance

Breeding habitats	Total number of habitats (n)	Number of habitats with vector immatures (%)	Number of habitats with any mosquito breeding (%)	Number of unapproachable habitats (%)
OHT (cement)	457	79 (17.29)	81 (17.72)	23 (5.03)
OHT (synthetic/fibre tanks)	414	20 (4.83)	22 (5.31)	91 (21.98)
Well	38	13 (34.21)	19 (50)	1 (2.63)
Stored water containers^a^	49	1 (2.04)	23 (46.94)	1 (2.04)
Sumps/underground tanks	17	1 (5.88)	1 (5.88)	0
Cisterns^b^	158	5 (3.16)	27 (17.09)	3 (1.9)
Inside tank (IST)	4	0	0 (0)	0

^a^Plastic pots, plastic containers, mud pots etc

^b^Transient breeding habitats like barrels or drums

### Influence of socio-demographic factors on the presence/absence of malaria

It was found that neither age nor gender significantly associated with malaria incidence/prevalence. However, presence of malaria cases were found to be significantly associated with the occupation (*χ*^2^ value = 3.898, p = 0.048). Out of 2471 individual subjects, 1540 were not staying in the house during day time and had gone either to work place or to an academic institution. Also, 65 of 1540 (4.2%) had malaria while 1475 of 1540 (95.8%) of them did not. Further, within 931 people who stayed at home, 25 of 931 (2.7%) had malaria while 906 (97.3%) did not have malaria.

When the influence of household factors on the malaria cases was analysed, it was observed that, malaria cases were significantly associated with the number of people residing in a particular household (*χ*^2^ value = 18.772, p < 0.001). Among 227 houses where ≤ 3 people resided, 9 (4%) had malaria and among 405 houses where more than 3 people resided, 62 (15.3%) had malaria. Furthermore, the type of roof structure was found to have significant association with the malaria cases (*χ*^2^ value = 12.506, p = 0.002). Out of 64 thatch roofed houses, 50 (78.1%) did not have any malaria patients, while 14 (21.9%) had malaria. Among the other structures, 10 (83.3%) out of 12 tiled roofed houses, did not acquire malaria infections whereas, 2 (16.7%) houses reported malaria. Further, 60 (76.9%) out of 78 asbestos roofed houses, did not have any malaria patients, while 18 (23.1%) had malaria. Out of 473 concrete roofed houses, 37 (8.0%) houses had malaria whereas, 436 (92.2%) did not suffer from malaria. It was observed that the concrete roofed houses were protective in terms of malaria infection with very limited vents/access to mosquitoes. Also, presence of a separate kitchen was significantly associated with the malaria prevalence in a household (*χ*^2^ value = 12.506, p = 0.002). Out of 632 houses, 450 had a separate kitchen, indicating better housing facilities. However, 41 (9.1%) out of 450 houses had malaria while 409 (90.9%) did not have malaria. Further, among the 182 houses without a separate kitchen, 30 (16.5%) had malaria. The usage of mosquito repellents, application of larvicide or fogging operation or animals in the proximity of households did not show any significant association with the presence/absence of malaria.

### Influence of breeding habitats on the presence/absence of malaria in a household

The influence of breeding habitats on the presence or absence of malaria cases was analyzed. It was found that the presence of inside tank (IST), stored water tanks, sump or wells did not significantly associate with malaria cases of a particular household. However, presence of overhead tanks was significantly associated with the presence of malaria cases (*χ*^2^ value = 15. 907, p < 0.001). It was observed that, 38 (8.2%) out of 463 houses with OHTs, were having malaria, while 425 (91.8%) did not report any malaria infection. Interestingly, 33 (19.5%) out of 169 houses without OHTs, had malaria cases. In addition, there was significant difference in the distribution of OHTs between the houses of asbestos, concrete, thatched and tiled roofed structures (*χ*^2^ value = 232.480, p < 0.001). Among 632 houses, 463 (73.2%) houses were having OHTs, 7 (1.5%) in thatched, 6 (1.3%) in tiled, 30 (6.5%) in asbestos and 420 (90.7%) in concrete roofed houses. 21.98% of the overhead tanks (synthetic/fibre tanks) were observed to be unapproachable (Table 2). The presence of cisterns was significantly associated with the presence of malaria cases (*χ*^2^ value = 8.365, p = 0.04). Out of 34 houses with cisterns, 9 (26.5%) were having malaria cases, while 25 (73.5%) did not report any malaria infection. Out of 598 houses without cisterns, 62 (10.4%) had malaria cases. Similarly, the presence of vector immatures in a particular household was significantly associated with malaria cases (*χ*^2^ value = 8.232, p = 0.04). Only 3 (3.0%) out of 101 houses with vector immatures, reported malaria. In contrast, 68 (12.8%) out of 531 houses without vector immatures, had malaria cases reported. The number, presence/absence of any breeding habitats in general, or whether the household had any unapproachable breeding habitat did not show any significantly association with malaria cases of a household. Nevertheless, presence of mosquito breeding (irrespective of species) was found to be significantly associated with the malaria cases of a household (*χ*^2^ value = 4.524, p = 0.033). A total of 143 houses had mosquito breeding at the time of survey, out of these, 9 houses (12.7%) reported malaria cases. Surprisingly, out of 489 houses without any mosquito breeding, 62 households (87.3%) reported malaria cases.

## Discussion

### Relationship between various attributes and malaria incidence/prevalence

Random sampling method was selected for the survey since it is the appropriate form of probability sampling as each member of the population has an equal and known chance of being selected. In Kenya, random mode of sampling has been done to find out the malaria prevalence in adults [9]. When the age group and gender was analysed, it did not show any significant association with malaria. However, presence of malaria was found to be significantly associated with occupation/vocation. The above result strengthens the presumption of outdoor biting since the working people are likely to leave home early and return back late, thus are more prone to the outdoor vector bites. Another possibility is that, the people who had returned after a tiresome day of work may unknowingly take a deep sleep unaware of the vector bites as reported elsewhere [10]. Frequent human travel for occupational purposes plays a major role in the establishment and maintenance of transmission [11]. It was also reported that livelihood practices were the major social determinants that influence malaria acquisition in central Tanzania [12]. Nevertheless, in the present study there was no observation of gender wise difference in malaria acquisition. On the other hand, women because of their occupational nature and lack of access to information were regarded as vulnerable population in terms of malaria [13]. Malaria infection was found to be more with increase in number (> 3) of household members. Similar results were also observed in studies from Madhya Pradesh, India [6]. It is quite possible that as the number of residents/inhabitants increases, the olfactory cues for the mosquitoes become stronger and are prone to increased number of vector bites [14].

Further, structure types with varied materials were found to influence the presence of malaria in a statistically significant way and residents living in concrete roofed houses were protected against malaria whereas, asbestos and thatched roof houses provided the least protection. While thatch roofs provide open eaves, asbestos roofs too provide enough entry points at the places where the roof joins the walls due to its wavery/parabolic shape. This was in concordance to the findings elsewhere, where poor quality of housing was reported as a major social determinant of malaria [13]. Residing in the highest quality houses reduced vector numbers [15]. Earthen roof as well as open eaves has been reported to be associated with increase in malaria risk [16]. However, in western Kenyan highlands, open eaves and uncovered windows did not appear to have an effect on the malaria incidence/prevalence [17].

It was observed that, 396 (88%) people with separate kitchen were living in concrete houses, while the concrete houses were observed to have less malaria incidence/prevalence. The presence of a separate kitchen was significantly associated with reduced malaria prevalence in the present study and similar results were also reported from Ethiopia where absence of separate kitchen was shown to be associated with increased malaria risk [18]. However, findings from western Kenyan highlands showed that separate kitchen had been reported to be related to higher risk of malaria incidence [7, 17]. It is well known fact that presence of a separate kitchen is likely to reflect their sound financial status capable of constructing separate rooms with better personal protection methods from vectors.

Furthermore, the presence of OHTs was found to show significant association with less number of malaria cases. It also reflects the fact that they belonged to better-protected houses, predominantly concrete roofed structures on which an overhead tank can be constructed or placed (like fibre tanks). It is observed that concrete houses are better protected against mosquito bites and therefore, malaria. In Kenya, concentration of breeding habitats was positively associated with malaria incidences [19], and proximity to the breeding sites increased the risk [20]. In contrast, the presence of cisterns was found to be significantly associated with more number of cases. Cisterns were numerous/aplenty and observed co-inhabitation of *An. stephensi* along with other mosquito species [5] and contributing predominantly to the abundance of *Culex* and *Aedes* species of mosquitoes [21].

It was interesting to note that, 77.92% of patients were having at least one type of breeding habitat in their house. However, the presence of any breeding habitat did not significantly associate with the malaria of a particular household. This poses a question against the common assumption that presence of water storage habitats increases the possibility of malaria acquisition. In Peru, houses with multiple cases were often located near a source of water [16]. Other confounding factors like outdoor biting or occupation leading to work/travel at night plays a crucial role in malaria incidence/prevalence. Interestingly, the presence of vector immatures and mosquito breeding was significantly associated with less number of malaria. Although prevalence in a year cannot be correlated with the recent scenario of vector/mosquito breeding, it reflects the potentiality of that habitat in contributing vector abundance and thereby malaria transmission. All the accessible breeding habitats in the study site are scheduled to receive routine weekly larviciding [5] and if the intervention is > 80% on weekly basis there is less chance of these habitats in contributing to adult vector density. In particular, this indicates that vector/mosquito breeding in a particular household may not be considered as the main indicator of the malaria risk since vector distribution and acquisition of malaria infection in an area can be affected by many other confounding factors.

The usage of repellents was not found to influence the presence or absence of malaria cases, similar to the results of study communities carried out in Ghana where application of mosquito coils did not reduce the incidence of malaria [22]. Vaporizers were reported to be the main repellents used, and was associated with higher socio-economic status (SES) in households in Chennai whereas; usage of coils was greater in the lower SES strata. Repellent use was associated with less malaria in a clinic study done in Chennai, though it was not reflected in the surveys [23]. However, since mosquito coils produce smoke, their deterrent effect could be the reason for vectors to bite outside during the early part of the night.

The study site with its dense population receives drinking water supply from Chennai Metropolitan Water Supply and Sewerage Board (CMWSSB). The OHTs, which are the potential breeding habitats, are numerous with an exponential increase on yearly basis. Earlier, cemented tanks used to be common and have been slowly replaced by synthetic fibre tanks, which are readily available in the market. 36.4% of the total breeding habitats were synthetic fibre OHTs, which covered 16.8% of the vector breeding habitats, encountered. The majority of the vector breeding habitats could be approached for inspection for survey with the access of step stones or ladders, and therefore, the vector control personnel could treat these habitats with their staff [5]. However, the present study also revealed the presence of synthetic water tanks (22%) including public distribution ones along the margin of the roads, which cannot be accessed as they are devoid of ladders or step stones. Further, 5.03% of cement overhead tanks were observed to be unapproachable. Hence, these habitats are less likely to be treated with larvicides and thus may probably act as efficient, undisturbed breeding ground for vector mosquitoes. In this context, the abundance of overhead tanks in the study area and the vector breeding preference has been reported earlier [5]. Since the country is set for ‘Malaria free India’ with an aim to eliminate malaria by 2030, necessary measures to tackle vector-breeding habitats are very essential to curtail transmission and to drive down malaria burden. In areas with slum settlement, a few breeding habitats are kept as public distribution water sources for many households and treatment of such habitats needs to be routine [7], which would be a recurring expenditure for the exchequer. Instead, long-term permanent solution of habitat manipulation by mosquito proofing overhead tanks or by replacing the existing ‘flap type open lid’ with ‘screw type lid’, would drastically reduce the vector breeding and minimize the need of frequent surveillance of such habitats. Besides, there could be a possibility of vector immatures becoming tolerant/resistant to the approved operational dosage of larvicide [5].

Implementation of culturally appropriate, sustainable, and effective interventions is crucial for the success of vector control strategies. Since, India stands next to Africa in the number of malaria cases with an observed increase of cases in urban areas, stringent measures of surveillance followed by control and follow up strategies have to be executed and monitored to reduce the disease burden [24, 25]. The present survey revealed many factors, which influenced the malaria incidence/prevalence in the study site in a significant way. The proportion of malaria cases in the study population (3.64%) did not represent the actual incidence/prevalence of malaria (18.6%) during 2014 [26]. Thus, it was assumed that, the study results might not reflect the true picture of the study site. Further, absence of significant association with many parameters indicates the presence of many confounding factors like travel and spatial heterogeneity, which affects the malaria prevalence in the study site. However, the present study can act as a reference in similar urban setting and periodic evaluation of the efficacy of the operational anti-vector control measures are extremely important for achieving the target of eliminating malaria.

The limitations of the present study is that it was undertaken in a malarious urban slum in Besant Nagar, Chennai, which is a part of Chennai and need not necessarily represent Chennai as a whole. The environmental parameters (temperature and relative humidity), of the study site has not been included in the present study.

## Abbreviations

OHT: overhead tank
UMS: Urban Malaria Scheme
IST: inside tank
SES: socio-economic status
CMWSSB: Chennai Metropolitan Water Supply and Sewerage Board
